# High glucose induces trafficking of prorenin receptor and stimulates profibrotic factors in the collecting duct

**DOI:** 10.1038/s41598-021-93296-4

**Published:** 2021-07-05

**Authors:** Venkateswara R. Gogulamudi, Danielle Y. Arita, Camille R. T. Bourgeois, Justine Jorgensen, Jing He, William C. Wimley, Ryosuke Satou, Alexis A. Gonzalez, Minolfa C. Prieto

**Affiliations:** 1 Department of Physiology, School of Medicine, Tulane University Health Sciences Center, 1430 Tulane Avenue, New Orleans, LA 70112 USA; 2 Department of Biochemistry, Tulane University School of Medicine, New Orleans, USA; 3 Instituto de Química, Pontificia Universidad Católica de Valparaíso, Valparaiso, Chile; 4 Hypertension and Renal Center of Excellence, Tulane University School of Medicine, New Orleans, LA USA

**Keywords:** Biological techniques, Physiology

## Abstract

Growing evidence indicates that prorenin receptor (PRR) is upregulated in collecting duct (CD) of diabetic kidney. Prorenin is secreted by the principal CD cells, and is the natural ligand of the PRR. PRR activation stimulates fibrotic factors, including fibronectin, collagen, and transforming growth factor-β (TGF-β) contributing to tubular fibrosis. However, whether high glucose (HG) contributes to this effect is unknown. We tested the hypothesis that HG increases the abundance of PRR at the plasma membrane of the CD cells, thus contributing to the stimulation of downstream fibrotic factors, including TGF-β, collagen I, and fibronectin. We used streptozotocin (STZ) male Sprague–Dawley rats to induce hyperglycemia for 7 days. At the end of the study, STZ-induced rats showed increased prorenin, renin, and angiotensin (Ang) II in the renal inner medulla and urine, along with augmented downstream fibrotic factors TGF-β, collagen I, and fibronectin. STZ rats showed upregulation of PRR in the renal medulla and preferential distribution of PRR on the apical aspect of the CD cells. Cultured CD M-1 cells treated with HG (25 mM for 1 h) showed increased PRR in plasma membrane fractions compared to cells treated with normal glucose (5 mM). Increased apical PRR was accompanied by upregulation of TGF-β, collagen I, and fibronectin, while PRR knockdown prevented these effects. Fluorescence resonance energy transfer experiments in M-1 cells demonstrated augmented prorenin activity during HG conditions. The data indicate HG stimulates profibrotic factors by inducing PRR translocation to the plasma membrane in CD cells, which in perspective, might be a novel mechanism underlying the development of tubulointerstitial fibrosis in diabetes mellitus.

## Introduction

In the kidney, the renin-angiotensin system (RAS) is activated during diabetes mellitus and contributes to the development of renal tissue injury^1–4^. Given that in diabetic patients the treatment with angiotensin converting enzyme inhibitors (ACEi) and angiotensin II (Ang II) type 1 receptor (AT1R) blockers only slow down, rather than halts, the progression of renal injury^5^, the mechanistic role of the intrarenal RAS in the pathogenesis and progression of diabetic nephropathy (DN) remains unclear. It has been suggested that tubulointerstitial fibrosis correlates better with DN than glomerular remodeling due to the impact in the progression of chronic kidney disease and kidney failure^6–7^.

The (pro)renin receptor (PRR) is a multifunctional receptor that has the ability to transduce signaling pathways related to tissue fibrosis in response to its agonists, prorenin and renin^8–9^. Activation of PRR stimulates mitogen-activated protein kinase (MAPK), extracellular regulated kinases 1/2 (ERK 1/2)^10^. The activation of PRR in mesangial cells, podocytes, and proximal tubule cells have been implicated in the pathogenesis of diabetic nephropathy^8,11,12^, however, the underlying mechanism by which PRR contributes to the development of tubulointerstitial fibrosis in the diabetic kidney remains unclear.

Hyperglycemia is a major factor in the induction of DN^13,14^. The elevated glucose levels in the diabetic kidney display numerous effects, including damage of mesangial cells^15,16^, as well as tubulointerstitial fibrosis^17,18^, due to oxidative stress^19^, accumulation of glycosylated products^20^, and increased cytokines^21^, High glucose (HG) contributes to the activation of intrarenal RAS^22^, In type 1 diabetic Sprague–Dawley rats, the principal cells of the CD are the main source of prorenin^3,23,24^, it is likely that local PRR activation by prorenin during HG conditions contributes to the development of tubulointerstitial fibrosis. In the present study, we tested the hypothesis that HG increases the abundance of PRR at the plasma membrane of the CD cells, thus contributing to the stimulation of downstream fibrotic factors, including TGF-β, collagen I, and fibronectin. We first induced hyperglycemia in Sprague–Dawley rats using streptozotocin (STZ) treatment. After 7 days of induction, we primarily assessed in the kidney, renin and prorenin intrarenal protein levels, PRR transcript and protein expression levels, and PRR cell distribution in the CD. Then, we examined the effects of glucose on PRR in cultured M-1 cells treated with either HG or normal glucose (NG) in the presence and the absence PRR shRNA to knockdown its expression. High glucose increased PRR protein expression levels in the plasma membrane and cytoplasmic cell fractions, augmented PRR-dependent stimulation of downstream profibrotic factors, and intensified the physical interaction between PRR and prorenin, measured by fluorescence resonance energy transfer (FRET) assay.

## Materials and methods

### Rats and sample collections

The study was carried according to international guidelines and regulations for animal care along with ARRIVE guidelines and regulation for animal care. All the experimental protocols were approved by the Tulane Institutional Animal Care and Use Committee under protocol ID 581. Male Sprague–Dawley rats (12–14 weeks old from Charles River Laboratories, Wilmington, MA, USA) were cage-housed and maintained in a temperature-controlled room with a 12:12 h light–dark cycle, with free access to tap water and standard rat chow (RALSTON PURINA, St. Louis, MO, USA) for 7 consecutive days. Then, rats were divided randomly into two groups: Control: (C; n = 7; normoglycemic rats) and STZ (n = 9; hyperglycemic rats). Hyperglycemia was induced via IP administration of a single streptozotocin (STZ) injection (60 mg/kg body weight) dissolved in 0.1 m citrate phosphate buffer (pH 4.5)^25^. Control group received a vehicle saline solution injection. After either 72 h STZ or saline (vehicle) IP injection, tail blood samples were obtained from after 6–8 h fast and blood glucose levels were measured using a blood glucometer. Only rats with fasting blood glucose concentrations ≥ 300 mg/dl were included in the diabetic (STZ) group. No insulin therapy was required in any of the STZ rats during the experimental protocol. Plasma and 24-h urine samples were collected two days before STZ induction and six days after it. On day 7 after STZ induction, the rats were euthanized by conscious decapitation and blood and kidneys were harvested. Plasmas and urines were used to measure renin activity, angiotensinogen (AGT) concentrations, Ang II levels, and soluble PRR concentration. Creatinine was measured using serum and urinary creatinine was measured using Creatinine Analyzer 2 (BECKMAN COULTER, Inc., Fullerton, CA). Protein concentration was determined by Bradford method (BIO-RAD, Hercules, CA). The urine protein/urine creatinine ratio was obtained by dividing urine protein concentration (mg/dL) by urine creatinine (mg/dL). Kidneys were decapsulated, weighted, cross-sectioned, and renal cortices were dissected from inner medullas for determinations PRR transcripts by qRT-PCR and immunoblotting.

### Renin activity (PRA) and Ang II levels in plasma

Blood samples were collected into chilled tubes containing 5.0 mmol/L EDTA to quantify plasma renin activity (PRA). PRA levels were expressed as nanograms per milliliter per hour of generated Ang I. For plasma Ang II levels, blood samples were collected into tubes containing a cocktail inhibitor solution (5 mmol/L of EDTA, 20 μmol/L of pepstatin, 20 μmol/L of enalapril, 1.25 mmol/L of 1–10-phenanthroline, and 10 μmol/L of PMSF) to avoid in vitro formation and degradation of the peptide^26^.

### Angiotensinogen, renin, soluble PRR, and Ang II excretion in urine

Urinary concentration of AGT and sPRR were measured by ELISA kits (IMMUNO-BIOLOGICAL LABORATORIES IBL, Co, Fujioka, Japan, Cat. # IB08003 and 27782, respectively). Ang II concentrations in urine were determined by radioimmunoassay (RIA), as previously described^26^. Results are expressed in fmol/24 h of urine. Urinary renin content (URC) was determined by using modified protocols from the PRA assay [GammaCoat Plasma Renin Activity ^125^I RIA kit (DIASORIN, Stillwater, MN, USA)]. Urine samples were spiked with 1 μM synthetic renin substrate tetradecapeptide (RST, Sigma). The generated Ang I was determined by RIA. To exclude the effect of peptidases, identical urine samples-RST with the specific renin inhibitor WFML peptide (ANASPEC; Fremont, CA) were used as controls.

### Total renin and Ang II content measurements in the renal inner medulla

Ang II was extracted from renal cortices and medullas and assayed as previously described^26^.

### PRR detection by immunohistochemistry

One kidney from each rat was paraffin-embedded, sectioned at 3 µm, and then stained using immunoperoxidase technique. Specific PRR immunoreactivity was assessed using rabbit anti-PRR antibody (*Atp6ap2 gene*, 1:200 dilutions; SIGMA CHEMICAL Co, St Louis, MO; Cat. # HPA003156) and detected with a donkey anti-rabbit antibody (1:1000 dilution, Alexa Fluor, INVITROGEN, Carlsbad, CA).

### Renin, PRR, and TGF-β transcripts quantification by real time qRT-PCR

Total mRNA was isolated from M-1 cells or rat kidney samples using RNeasy Mini Kit (QIAGEN, Valencia, CA) according to the kit protocol. Total RNA was quantified using nano-drop system. Quantitative real-time RT-PCR (qRT-PCR) was performed using the TaqMan PCR system. Primer and probes sequences used for PRR amplification are previously described for rat^27^: 5′-ATCCTTGAGACGAAACAAGA-3′ (Sense), 5′-AGCCAGTCATAATCCACAGT-3′ (antisense), 5′-6-FAM-ACACCCAAAGTCCCTACAACCTTG-BHQ1-3′ (probe). Primers and probe for mouse PRR amplification were 5′-GGTGTTTGAAGACCTTTCAG-3` (sense), 5`-GCAGGTCAACCTCATTATTC-3`, 5′-6-FAM-TCCGTAAACGCCTGTTTCAAGAA BHQ1-3′ (probe). Primers and probe used for rat TGF-β was: 5′-TACCATGCCAACTTCTGTC-3′ (Sense), 5′-AAGGACCTTGCTGTACTGTGT-3′ (antisense) and 5′6FAM-CCCTACATTTGGAGCCTGGAC-BHQ1-3′ (probe). The mRNA levels were normalized against the expression level of rat/mouse compatible β-actin sequence using 5′-ATC ATG AAG TGT GAC GTT GA-3 (sense); 5′-GATCTTCATGGTGCTAGGAGC-3 (antisense); and 5–6-HEX-TCTATGCCAACACAGTGCTGTCTGGT-BHQ2-3 (fluorogenic probe). Results were presented as a ratio between the levels of mRNA of the interest gene against β-actin (“housekeeping” gene).

### Immunoblotting analyses

Protein samples were electrophoretically separated on a precast NuPAGE 10% Bis–Tris gel (NOVEX) at 200 v for 45 min followed by semi-dry transference to a nitrocellulose membrane using iBlot (INVITROGEN, Carlsbad, CA, USA). Blots were blocked with Odyssey blocking buffer (LI-COR BIOSCIENCES, Lincoln, NE, USA) at RT for 3 h, incubated overnight with specific primary antibody at 4 °C, followed by the incubation with the corresponding secondary antibodies donkey anti-rabbit or anti-mouse (1:15,000 dilutions), at room temperature for 45 min and analyses by normalization against β-actin, used as a loading control. Renin and prorenin protein levels were quantified using the rabbit anti- renin/prorenin antibody (Cat. # sc-22752; SANTA CRUZ, CA). PRR protein levels were detected using a polyclonal rabbit anti-PRR (*Atp6ap2*, 1:200, SIGMA-ALDRICH) that recognizes the intracellular segment and the ectodomain^27^. Furin was detected using rabbit anti-furin (Ab3467, 1:500, ABCAM, Cambridge, UK). Activation of ERK pathway was determined by using mouse anti-phospho-p44/42 ERK1/2 (Thr202/Tyr204) antibody (Cat. # 91065, CELL SIGNALING TECHNOLOGY, Beverly, MA), and a rabbit anti total ERK (Cat. # 9122, CELL SIGNALING TECHNOLOGY, Beverly, MA). Results were normalized to β-actin, which was used as a housekeeping protein (see supplementary information).

### M-1 cell culture and reagents

The M-1 cell line derived from the CD of mice^28^, was purchased from American Type Culture Collection (ATCC, Manassas, VA). M-1 cells are composed by principal and intercalated cells, this cell model has been validated previously^29–31^. Prior to the experiments, M-1 cells were maintained in Dulbecco′s modified Eagle’s medium (DMEM)/F-12 medium (LIFE TECHNOLOGIES, Carlsbad, CA) containing 10% fetal bovine serum (FBS), growth factors (transferrin, insulin, and sodium selenite at physiological concentrations), 100 nmol/L dexamethasone and 5 mM glucose. When the cells reached approximately 70–75% confluence, they were dissociated using trypsin and split into 4-wells chambers and dishes for the trafficking assays.

### Trafficking assay of the prorenin receptor (PRR)

To determine whether HG induces the translocation of PRR from the cytosol to the cell surface we used trafficking assay in M-1 cells treated with either normal glucose (NG) or HG. Briefly, cultured cells were split into 10 cm dishes and incubated with culture media containing normal glucose (NG, 5.5 mM glucose + 19.5 mM mannitol) and high glucose (HG, 25 mM glucose) in supplement serum free media for 1, 6 h specified in each protocol. Abcam Plasma Membrane Isolation Kit (Ab65400; ABCAM, Cambridge) was used to extract the membrane proteins. Western blotting was performed using antibody anti-PRR (*Atp6ap2*, 1:200, SIGMA-ALDRICH) overnight at 4 °C, followed by the incubation with the secondary antibody donkey anti-rabbit (1:10,000), at room temperature for 45 min. Detection was accomplished using the Odyssey detection system (LI-COR BIOSCIENCES, Lincoln, NE) and protein expression was normalized against E-cadherin.

### Renin secretion to extracellular media

To examine renin secretion into culture media, M-1 cells were starved overnight without glucose, then treated with NG and HG at different time intervals. Supernatants were collected and 50-fold concentrated by using Amicon Ultra-4 (MILLIPORE, Carrigtwohill, IE). Briefly, 4 ml of culture supernatant was added to the ultra-filter device, and centrifugated to concentrate at 4,000 g for 15 min. Concentrated cell culture media was recovered, and immunoblotting analysis was completed as mentioned above by loading an equal volume of protein. Equal loading of proteins was confirmed by Ponceau red staining.

### PRR immunofluorescence (IF) and immunocytochemistry (ICC)

To further evaluate the translocation of the PRR from the cytosol to the cell surface we used IF/ICC. Cells were split to 4-wells ICC chambers and incubated with culture media containing NG or HG in supplement-free media (except P/S) for 1 h. After culture media removal, cells were fixed with 4% PFA in PBS (30 min, 4 °C), incubated with 1% Triton (4 min), fixed in 4% PFA in PBS (15 min, 4 °C), followed by sequential incubation with Signal Enhancer (INVITROGEN, CA) for 2 h at RT, and anti-PRR (*Atp6ap2*, 1:200 dilutions; Cat. # HPA003156; SIGMA-ALDRICH, MO, USA) overnight at 4 °C. The signals were detected with an anti-rabbit green secondary antibody at a 1:1500 dilution, for 45 min at RT. Cells were washed (2-3x) with PBS between steps. The slides were mounted with ProLong Gold with 4,6-Diamidino-2-phenylindole dihydrochloride (DAPI) for nuclei staining (INVITROGEN, CA). The images were obtained using a Nikon Eclipse-50i immunofluorescence microscope (Nikon Eclipse-50i, Japan) and were digitalized using the NIS-Elements (BR version 4.0 from NIKON, Japan). Cell membrane or cytosolic localization of PRR was further assessed by de-convolution of IF images.

### Assessment of the physical interaction between PRR and prorenin in M-1 cells treated with high glucose using FRET

To assess whether HG increases the physical interaction between PRR and prorenin in M-1 cells, we first used a fluorescence resonance energy transfer (FRET) assay by means of a Fluorometric Sensolyte 520 Mouse Renin Assay Kit (ANASPEC INC, Cat. # 72161) following the manufacturer’s protocol. Briefly, M-1 cells treated either with NG or HG for 1 h were cultured, and plasma membrane (PM) fractions were extracted using the membrane protein extraction kit (ab65400, ABCAM). Pellets of protein fractions were solubilized in homogenization buffer followed by incubation in 10 nM prorenin for 30 min at 37 °C. Ten nanomolar trypsin-activated prorenin was used as a positive control and renin inhibitor Ac-HPFV- (Sta)-LF-NH2 was used as a negative control. Fluorescence intensity was read using the Synergy 2 Multi-Detection Microplate Reader (BIOTEK, VT) before and after the addition of the substrate (angiotensinogen, AGT) for 110 min. For the kinetic measurements, excitation wavelength was 490 nm and emission recorded at 520 nm. The final fluorescence intensity value of 5-FAM/QXL versus time was plotted and the variations of the fluorescence intensities of NG and HG were compared to controls as an indicator of the amount of final product of the renin enzymatic activity. Values were corrected by the amounts of protein concentration quantified in the membrane fractions.

### PRR knockdown using transient transfections of shRNA-PRR

Silencing of PRR expression was performed using SureSilencing shRNA plasmid for Mouse Atp6ap2 (Cat. # KM26726, SABIOSCIENCE, QIAGEN, Valencia, CA) as described previously^8^. Cells 60% confluency were transfected 32 h before treatments. Cell viability after PRR knockdown (48 h) assessed by AlamarBlue Cell Viability Reagent (THERMOFISHER, Cat.# DAL1025) demonstrated no increases in cell death.

### Statistical analyses

Results are expressed as mean ± SEM. Grubb’s test was used to detect outliers in univariate data assumed to come from a normally distributed population and using a significance level of alpha = 0.05. These exclusion criteria were applied in experiments using more than 6 samples. Comparisons between groups were performed using One-Way ANOVA and Tukey’s post-test, when appropriate. P ≤ 0.05 values were considered statistically significant, with P = NS demonstrating no significance.

## Results

### STZ induced hyperglycemia after 7 days of administration

As shown in Table 1, the bodyweight of STZ rats decreased at 7d after STZ administration and was significantly less than that of the control rats. Overnight fasting blood glucose levels were significantly higher and plasma insulin levels were significantly lower in STZ-induced hyperglycemic rats than in control rats. Urine volume and protein excretion were higher in STZ rats compared with the control group, whereas urinary creatinine levels were lower in STZ rats than in controls.

**Table 1:** Physiological parameters of STZ rats on Day 7, body weight, blood glucose, plasma insulin, urine volume, urine protein concentration, urinary creatinine, urine protein/creatinine ratio.

	Control (n = 7)	STZ (n = 9)
Body weight (g)	506.9 ± 11.0	426.9 ± 16.7**
Blood glucose (mg/dL)	137.7 ± 9.3	427.7 ± 12.9***
Plasma insulin (ng/mL)	2.42 ± 0.6	0.05 ± 0.02***
Urine volume (mL/24 h)	20.6 ± 1.5	106.2 ± 17.5***
Urine protein (mg/dL)	73.8 ± 4.6	203.7 ± 9,7*
Urinary creatinine (mg/dL) Urine protein/creatinine ratio	124.2 ± 15.3 0.59 ± 0.01	31.7 ± 5.3*** 6.42 ± 0.23

Mean ± SEM, *P < 0.05, **P < 0.01, ***P < 0.00.

### STZ rats showed a marked increase of renin and prorenin peptides in renal tissues and augmented urinary renin activity

**Figure 1:**
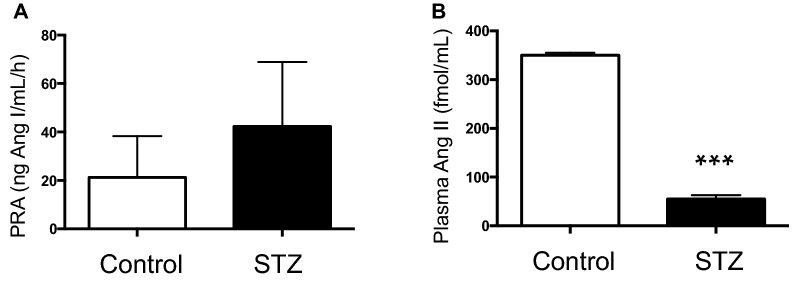
Changes in plasma renin and Ang II contents in streptozotocin (STZ)rats. Plasma renin activity (**A**) and plasma Ang II levels (**B**) in control (n = 7) and diabetic hyperglycemic rats (n = 9) induced by STZ administration for 7 d. *** P < 0.001 versus control group.

**Figure 2:**
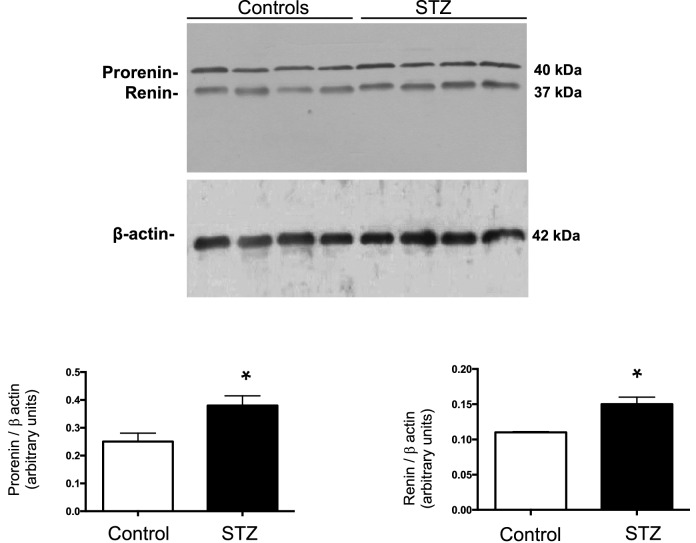
Prorenin and renin in the renal medulla. Representative immunoblots (controls n = 4 and STZ, n = 4) showing prorenin and renin protein levels in renal medullary tissues from control and STZ rats.

**Figure 3:**
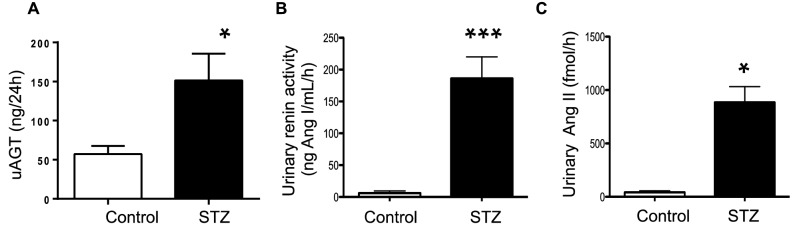
Urinary excretion of RAS components in the urine. Urinary angiotensinogen (AGT) (**A**), Urinary renin activity (**B**) and urinary Ang II levels (**C**) in control (n = 7) and STZ-induced diabetic rats (n = 9) *P < 0.05 versus control group.

After 7d of STZ administration, plasma renin activity in hyperglycemic rats averaged 42.32 ± 11.87 versus 21.28 ± 8.50 ng Ang I/mL/h (Fig. 1A). Ang II levels were markedly lower in plasma of STZ rats compared with control rats (STZ: 60.1 ± 5.6 vs. C: 358.2 ± 1.7 fmol/mL, *P* < 0.001) (Fig. 1B). Renin and prorenin proteins were both greater expressed in the renal medulla of STZ than in control rats (Fig. 2). AGT was increased in STZ rats (STZ: 57.18 ± 10.57 vs. C: 151.5 ± 34.18 ng/24 h, p < 0.022, Fig. 3A). Excreted urinary renin was also increased (STZ: 186.2 ± 33.91 vs. C: 6.22 ± 3.28 ng Ang I/mL/h, *P* < 0.01, Fig. 3B) as well as urinary Ang II (STZ: 884.2 ± 147.1 vs. C: 41.6 ± 14.3 fmol/h, *P* < 0.05) (Fig,. 3C).

### PRR expression is augmented in the renal medulla of STZ rats

**Figure 4:**
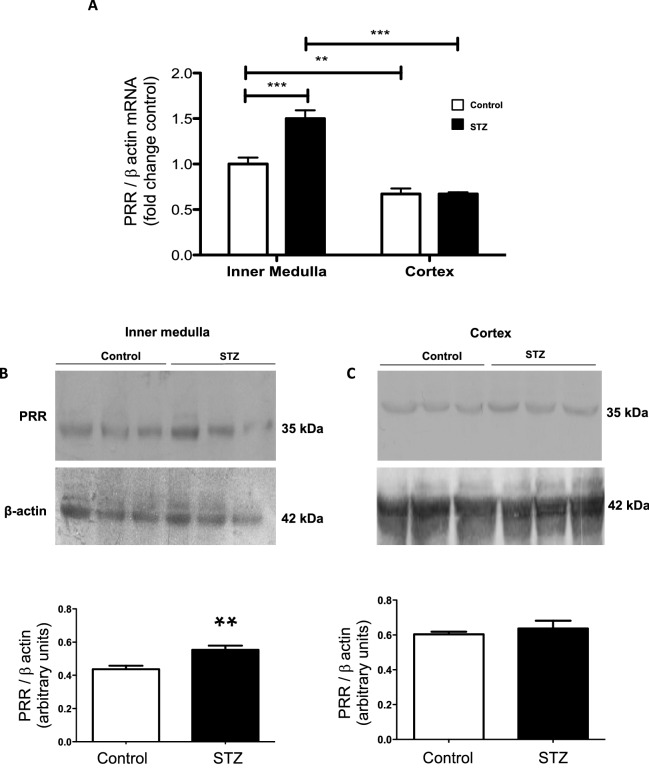
Prorenin receptor is increased in medullary tissues of diabetic rats. (**A**) PRR mRNA levels were augmented in the renal medulla in STZ rats. No differences were found in the cortex. (**B**) Representative immunoblot showing the induction of PRR protein levels in STZ rats as compared to controls. (**C**) No differences were observed in the renal cortex (n = 3). *P < 0.05, **P < 0.01, ***P < 0.001.

**Figure 5:**
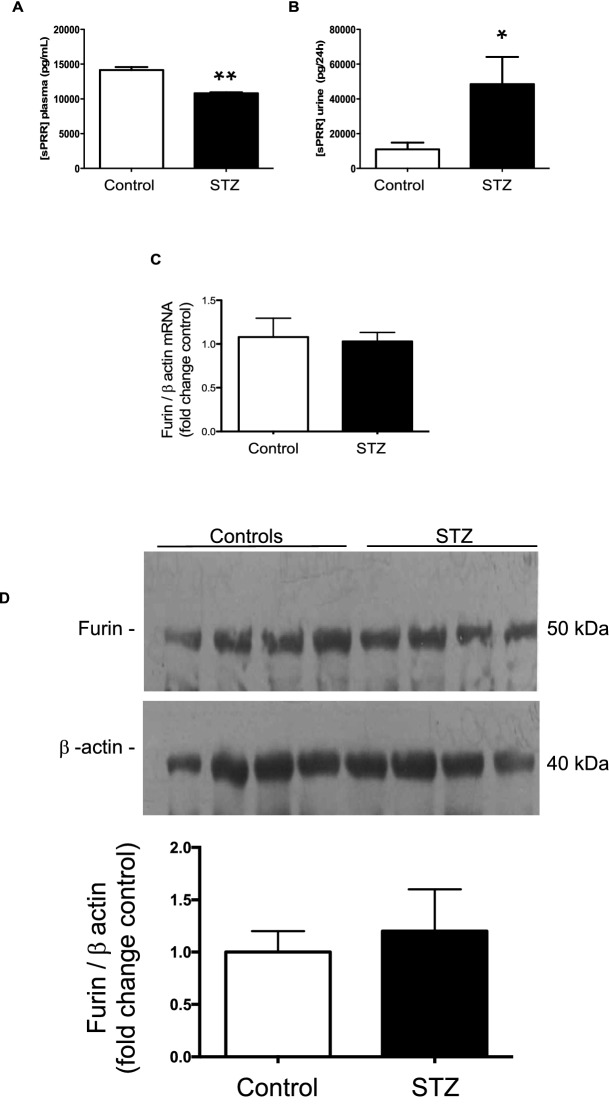
Soluble PRR (sPRR) measurements in plasma and urine. The sPRR was decreased in plasma from STZ rats (**A**), while in the urine sPRR was augmented (**B**). Furin mRNA (**C**) and protein (**D**) levels (n = 4) showed no significant changes in STZ rats compared to control rats (**C**). *P < 0.05, **P < 0.01.

Figure 4A–C shows the renal PRR mRNA and protein levels. PRR mRNA was markedly increased in the renal medulla of STZ rats (fold change of control: STZ: 1.50 ± 0.09 vs. C: 1.0 ± 0.07, *P* < 0.001) (Fig. 4A). The protein expression levels of the full-length form of the PRR were greater in STZ rats than in control rats (PRR/β-actin protein ratio: STZ: 0.55 ± 0.03 vs. C: 0.44 ± 0.02, *P* < 0.01, Fig. 4B). In the cortex, PRR expression was not altered in STZ rats (Fig. 4C). Because soluble form of the PRR (sPRR) also interacts with prorenin ^8,27^ we evaluated sPRR levels in urine and plasma. Although diabetic rats demonstrated significantly lower levels of sPRR in plasma (STZ: 10,814 ± 138 vs. C: 14,146 ± 426 pg/mL, *P* < 0.001) (Fig. 5A), urinary sPRR levels were higher when compared with control rats (STZ: 48,402 ± 15,667 vs. C: 10,897 ± 3,969 pg/24 h, *P* < 0.05) (Fig. 5B). Because furin is one of the intracellular serine proteases responsible for sPRR formation ^32,33^, we evaluated the mRNA (Fig. 5C) and protein (Fig. 5D) levels of furin in whole kidney samples. However, no differences were found in kidney tissues between the two groups.

## STZ rats showed increased apical membrane distribution of PRR in the collecting duct

**Figure 6:**
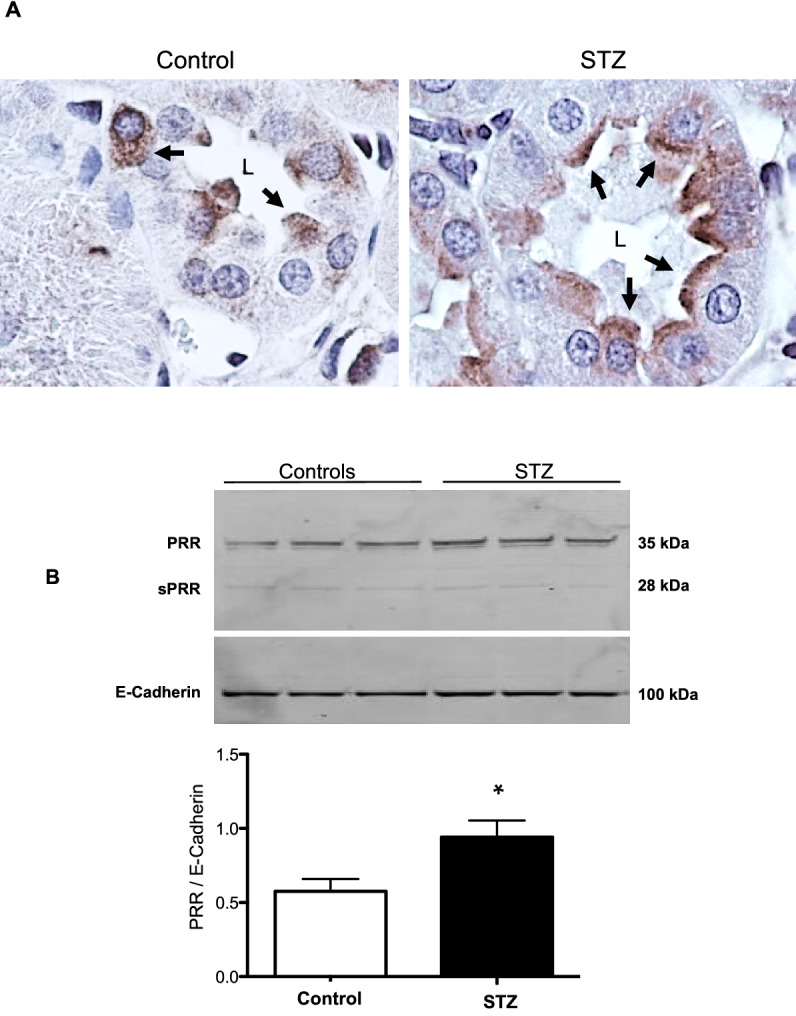
PRR is translocated to the cell plasma membrane in collecting ducts of STZ rats. (**A**) Representative image of PRR immunohistochemistry in medullary kidney slides of control and STZ diabetic rats. As observed, PRR is mostly observed on the apical side of the collecting duct cells (arrows) in STZ rats, while in control rats PRR is present more diffusely in the cytoplasm. (**B**) Representative immunoblots (n = 3) showing the increase of PRR abundance in the plasma membrane fractions in medullary tissues of STZ rats. E-cadherin was used as a constitutive protein for plasma membrane fractions. *P < 0.05 versus control rats.

Immunohistochemistry was performed to determine whether HG conditions cause changes in the specific localization of the PRR in the CD. In STZ rats, PRR was detected at the luminal side of the plasma membrane in renal medullary CD cells, while in control rats, PRR was homogeneously distributed within each cell (Fig. 6A, arrows). Enriched plasma membrane fractions from control and STZ rats were extracted from renal medullary tissues. We found a higher abundance of PRR at the plasma membrane of renal medullas from STZ rats compared to control rats (STZ: 0.94 ± 0.11 vs. C: 0.57 ± 0.08, *P* < 0.05) (Fig. 6B).

### TGF-β mRNA is augmented in the renal medulla, but not cortex, in STZ rats

**Figure 7:**
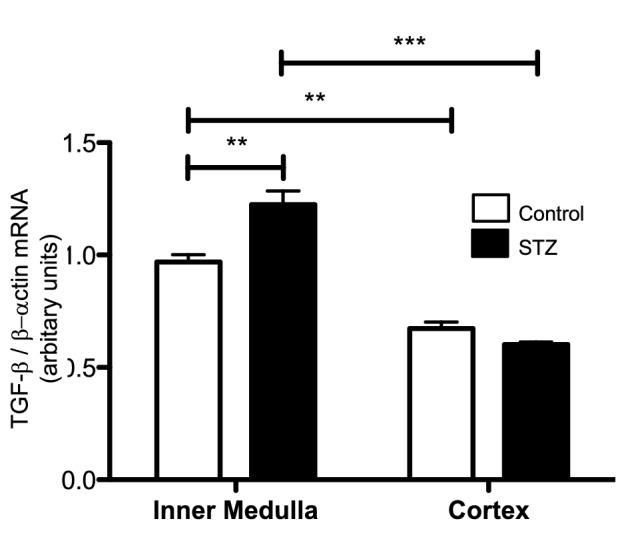
Quantitative RT-PCR in medulla and cortex of control and STZ rats showed the induction of TGF-β in the renal medulla but not in cortical tissues (n = 4). *P < 0.05, **P < 0.01.

Figure 7 shows that expression of TGF-β mRNA in the renal medulla was significantly higher than in the cortex and was markedly greater in renal medullae of STZ rats than in control rats (STZ: 1.22 ± 0.06 vs. C: 0.97 ± 0.03 TGF-β/β-actin mRNA ratio, *P* < 0.01).

### Glucose induces PRR trafficking to the plasma membrane and facilitates the interaction between PRR and prorenin in M-1 cells

**Figure 8:**
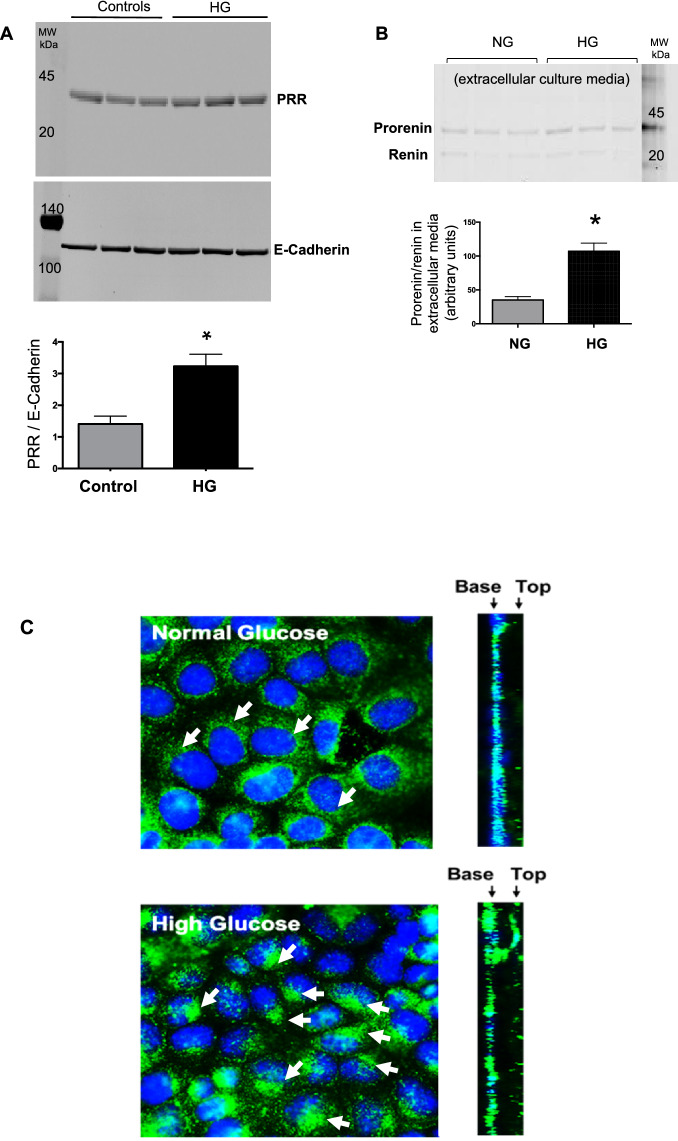

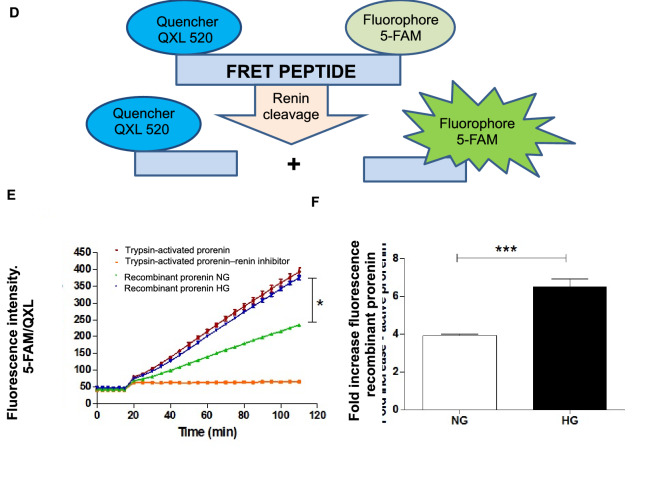
In vitro studies in M-1 cells showing the effect of high glucose conditions on PRR translocation to the cell plasma membrane. (**A**) Membrane fractions from M-1 cells showed increased levels of PRR after 6 h of HG treatment (n = 3). (**B**) Representative image of an immunoblot from supernatant cell culture media after 50-fold concentration showing increased prorenin and renin abundance after HG treatment (n = 3). (**C**) PRR immunofluorescence in NG and HG conditions. In NG conditions the PRR is localized mainly in the perinuclear area (arrows). After 1 h of HG incubation, the PRR showed high abundance at the apical side of the cell (arrows and insert at right). (**D**–**F**) Angiotensinogen fluorescence resonance energy transfer (FRET) experiments. Pellets of protein fractions were incubated with 10 nM prorenin for 30 min at 37 °C. Ten nanomolar trypsin-activated prorenin was used as a positive control and renin inhibitor Ac-HPFV-(Sta)-LF-NH2 was used as a negative control. Fluorescence intensity was read using the Synergy 2 Multi-Detection Microplate Reader (BioTek) before and after addition of the substrate (angiotensinogen, AGT) for 110 min. The final fluorescence intensity value of 5-FAM/QXL versus time was plotted and the variations of the fluorescence intensities of NG (green line) and HG (blue line) were compared to positive (trypsin activated prorenin, brown line) and negative control (in the presence of renin inhibitor, orange line), as an indicator of the amount of final product of the renin enzymatic activity. Values were corrected by the amounts of protein concentration quantified in the membrane fractions (n = 3). *P < 0.05; ***P < 0.001. (**D**) was drawn by first author using powerpoint software MICROSOFT OFFICE 2019 (https://www.office.com).

M-1 cells were incubated with HG media for 1, 6 h. HG treatment in M-1 cell increased PRR protein abundance in the plasma membrane at 6 h (HG: 3.23 ± 0.38 vs. NG: 1.410 ± 0.25, PRR/E-cadherin protein ratio P < 0.05) (Fig. 8A). No differences were found at 1 h (data not shown). Prorenin and renin secretion to the cell culture media was induced as early as 1 h of HG incubation (HG: 107 ± 12 vs. 35 ± 5, pixel intensity/region of prorenin plus renin band, P < 0.05, (Fig. 8B). Under normal glucose conditions, PRR immunofluorescence showed perinuclear area localization (Fig. 8C). After incubation with HG (1 h), the immunoreactivity was spread diffusely inside the cell, with increased localization at the apical surface of the cells (Fig. 8C right insert). To further examine whether HG enhances the physical interaction between PRR and prorenin, we used FRET assay using plasma membrane fractions from M-1 cells in NG or HG conditions. Figure 8D shows the AGT-FRET experiment methodology and the fluorescence intensity measured after the addition of substrate AGT (Fig. 8E). The intensities were normalized by the same amounts of protein amount extracted. Quantification of FRET data indicated that even in the presence of similar amounts of extracted plasma membrane fractions from NG and HG treated M-1 cells, there was an increase in renin activity during HG conditions (Fig. 8F).

### Induction of TGF-β, fibronectin and collagen I by high glucose is dependent on PRR

**Figure 9:**
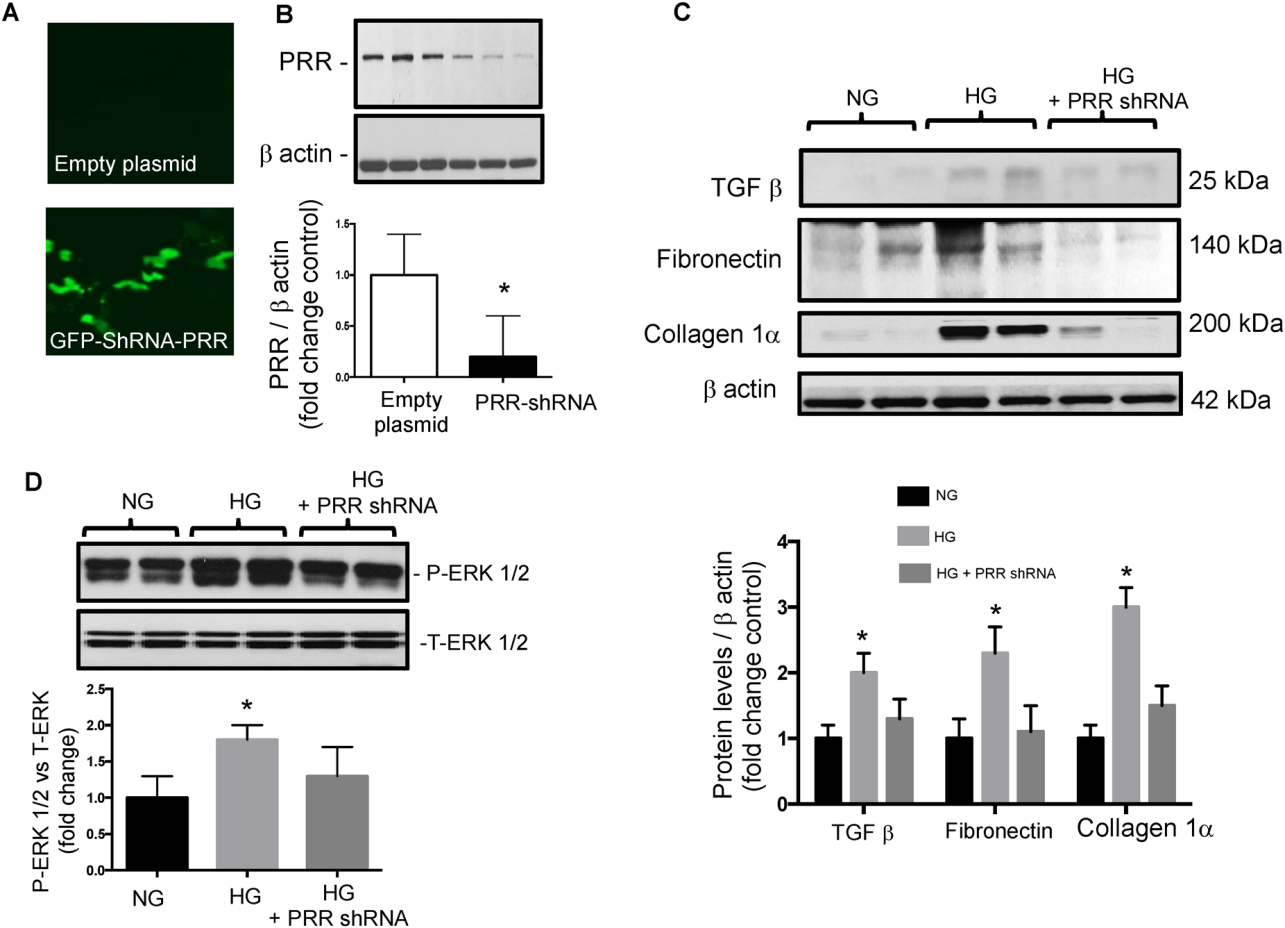
High glucose increases ERK pathway and fibrotic factors via PRR in M-1 cells. (**A**) M-1 cells were transfected with a green fluorescent protein (GFP)-shRNA PRR GFP or an empty plasmid as a control. (**B**) Representative immunoblot showing that shRNA PRR reduced PRR protein levels by 83% as compared to cells transfected with an empty plasmid (n = 7). (**C**) Representative immunoblots and densitometric analysis of TGF β, collagen 1α and fibronectin protein levels after 6 h of incubation with normal (NG), high glucose (HG) and HG conditions plus PRR knockdown (n = 4). No differences in TGF-β, collagen 1α and fibronectin protein levels were observed in NG conditions in cells transfected with shRNA-PRR as compared to controls (data not shown). The β-actin was used as a housekeeping protein. (**D**). Phospho-ERK (P-ERK) intensity versus total ERK (T-ERK) represented as fold change of control demonstrated the increased ratio between P-ERK and T-ERK in HG conditions (10 min) that was partially prevented by shRNA PRR transfections (n = 4). *P < 0.05 versus NG group.

To determine whether the induction of profibrotic proteins depends on PRR, we performed PRR knockdown experiments by transfecting the M-1 cells with GFP-PRR-shRNA 32 h before treatments (Fig. 9A) to reduce the protein levels of PRR as previously described^8^. PRR protein levels were reduced by near 83% after 32 h (1.1 ± 0.4 vs. 0.2 ± 0.4, Fig. 9B). HG incubations for 6 h promoted the induction of TGF-β, fibronectin and collagen 1α protein levels. The induction in all three profibrotic markers (TGF-β: 2.1 ± 0.3 vs. 1.1 ± 0.2; fibronectin: 2.3 ± 0.4 vs. 1.0 ± 0.2; collagen 1α: 3.1 ± 0.3 vs. 0.9 ± 0.1, P < 0.05) was prevented by PRR-shRNA transfection (Fig. 9C). Induction of phospho-ERK1/2 was partially prevented by PRR-shRNA transfection (Fig. 9D).

## Discussion

In the present study, we report that SD rats with STZ-induced hyperglycemia for 7 days exhibit augmentation of PRR, and prorenin and renin proteins expression in the CD of the kidney. These changes are accompanied by augmented urinary excretion of AGT renin, Ang II, sPRR. We further showed evidence that HG induces PRR translocation to the plasma membrane of M-1 cells, which increases the physical interaction between PRR and prorenin, PRR-dependent activation of ERK pathway, and upregulation of downstream targets as TGF-β, fibronectin and collagen I.

The PRR is considered a multifunctional receptor^34^. As an accessory protein of the multi-subunit complex, vacuolar H^+^-ATPase (v-ATPase), PRR plays a key role in intracellular acidification^35,36^, autophagy^37^, and kidney development^38^. In the kidney, the expression of v-ATPase on the membranes of intracellular organelles and A-type intercalated cells^39^, emphasizes the relevance of PRR in the regulation of intracellular pH^36^. In addition, in vitro evidence indicates that PRR increases renin activity and fully activates prorenin^27,40^. These findings are further supported by in vivo data from different models of experimental hypertension demonstrating that PRR in the CD is required for the local formation of Ang II^41,42^. However, whether PRR binding to prorenin increases renin activity and fully activates prorenin in vivo, still remains controversial^43^.

**Figure 10:**
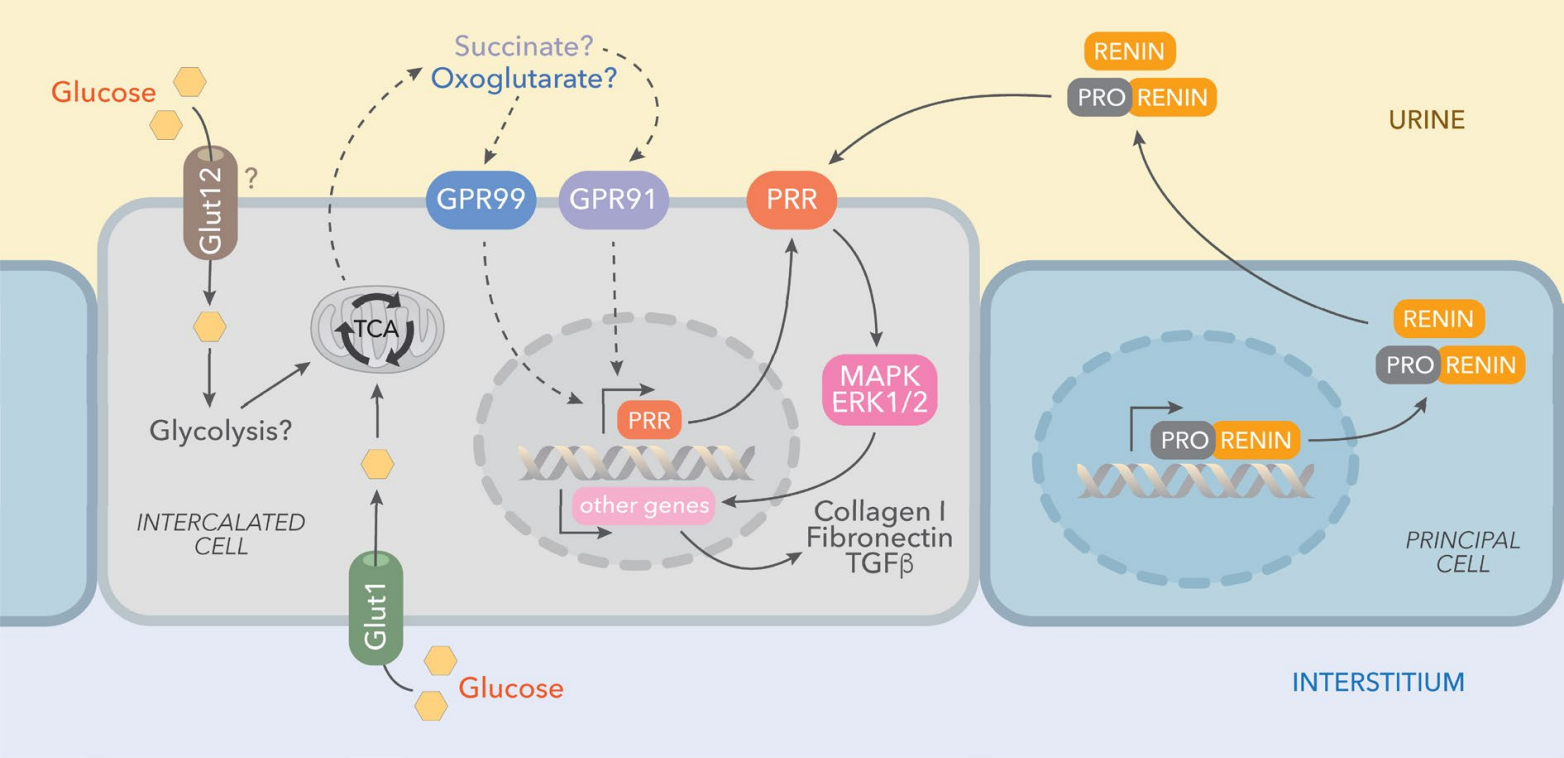
Working hypothesis representing the potential effects of high glucose on PRR cellular preferential distribution to the plasma membrane. In the collecting duct, the principal cell is the main source of prorenin in diabetes. Prorenin released to the lumen, binds PRR, thus leading to its activation and stimulation of MAPK/ERK1/2 intracellular pathway which is known to induce profibrotic factors in these cells. Upregulation of PRR is mediated by high glucose, but not osmolality. In this scenario, it is likely that GLUT-1 and GLUT-12 transporters contribute to the HG-dependent PRR trafficking through mechanisms including glycolysis and metabolic intermediaries such as succinate and alpha-ketoglutarate through their receptors.

Augmented PRR expression may play important pathophysiological roles in the development and progression of renal fibrosis during DM. Likewise, PRR mRNA and protein abundance are increased in the hearts of TGR[m(Ren2)-27] diabetic rats in association with diastolic dysfunction, myocyte hypertrophy, and interstitial fibrosis^44^. Furthermore, PRR protein levels are augmented in mesangial cells during HG conditions^45^ and in CD during diabetes^46^. In the present study, PRR protein levels were augmented in the renal inner medulla of STZ-induced hyperglycemic SD rats (Fig. 4), which are devoid of glomeruli and primarily contain CD. We detected a band of 35 kDa, which corresponds with the full-length PRR molecular form (Fig. 4). Along with the increased expression of PRR protein, the preferential apical distribution of PRR in the CD of STZ-induced hyperglycemic rats as compared to control rats suggests that HG also influences PRR cell distribution in the distal nephron segments. We also demonstrated that M-1 cells incubated with HG showed increased PRR protein expression in the plasma membrane fractions after 1 h and 6 h treatment compared with NG-treated cells (Fig. 8A,C). Kang et al. demonstrated that CD is the main source of prorenin in STZ-type 1 diabetic rats^3^. Therefore, our evidence demonstrating that in M-1 cells, HG increases mainly prorenin in the extracellular media (Fig. 8B), and stimulates PRR trafficking to the PM further supports our hypothesis that HG increases the physical interaction between prorenin and PRR in the CD. Our data is also supported by evidence demonstrates that glucose stimulates polarized translocation of v-ATPase to the apical plasma membrane in proximal tubular HK-2 cells^47^. This concept was further confirmed using FRET, a tool that allows examining the energy transfer between a donor and acceptor pair of fluorophores, thus quantifying molecular dynamics and the interactions between proteins. When the donor and acceptor fluorophores are in close proximity to each other, excitation of the donor results in detectable emission only from the acceptor. Using a fluorogenic renin substrate, AGT, containing the renin cleavage site at the Leu‐Val bond^48^, we demonstrated FRET-based assay or renin enzymatic activity in M-1 cells treated with HG. The activation of recombinant prorenin as shown by the increased renin activity in plasma membranes fractions as compared to those extracted from NG-treated cells (Fig. 8D,E,F) reflect the physiological significance of the effect of HG on the enhancement of physical interaction between PRR and prorenin, which might be of high relevance in diabetes. The underlying mechanisms involved in the regulation of PRR by HG have been studied in mesangial cells^45,49,50^, and might be related to glucose transport. The CD express GLUT transporters, particularly GLUT-1 and GLUT-12^51^ These glucose transporters may modulate HG-dependent PRR trafficking through mechanisms such as glycolysis and metabolic intermediaries such as succinate and alpha-ketoglutarate (Fig. 10) as suggested previously^52^, indeed it has been shown that in the collecting duct the GLUT transporters are upregulated during HG conditions^51^. Additional studies are necessary to elucidate the intrinsic mechanism involved in this process.

Activation of PRR promotes the induction of profibrotic genes^8,11^. However, elevations in Ang II, blood pressure, inflammation, and oxidative stress may also stimulate fibrotic factors^53–56^. The fact that binding of prorenin to PRR triggers intracellular signals linked to tissue fibrosis raises the possibility whether prorenin could be directly responsible for tissue fibrosis during the early phase of diabetes, this aspect should be further determined in additional studies by using collecting duct specific AT1 receptor knockout^57,58^. The PRR/MAPK/ERK1/2 pathway mediates the stimulation of TGF-β, fibronectin, and collagen leading to tissue fibrosis and inflammation^59,60^. In the present study, SD rats with STZ-induced hyperglycemia exhibited increased expression of medullary TGF-β along with augmented mRNA and protein levels of PRR in the renal inner medulla (Figs. 6, 7). Furthermore, HG treatment of M-1 cells not only increased PRR in the plasma membrane fractions, but also stimulated the expressions of TGF-β and fibrotic factors, including fibronectin and collagen (Fig. 9C). This effect was prevented by PRR knockdown. It should be noted that changes in the PRR and prorenin/renin observed during the very early phase of STZ- induced diabetes may let us evaluate in future studies whether these changes continue in later stages of the diabetic disease or in the presence of hyperglycemia.

In the kidney, hyperglycemia augments AGT in the proximal tubule, changes that are associated with high blood pressure and DN via stimulation of oxidative stress^61^. Inhibition of glucose transport via SGLT2 in the proximal tubule prevents intrarenal AGT upregulation. Although we did not measure blood pressure in our model, it is likely that during the late phase of the STZ-diabetic rat model, high levels of circulating prorenin^62^ augmented expression of renin in the collecting duct^3^ and AGT in the proximal tubule^2^ along with the increased abundance of PRR in the plasma membrane of the collecting duct cells may promote high blood pressure, renal tubular fibrosis^63^. Future studies are necessary to determine the progression of the blood pressure along the early and late stages of STZ-induced diabetic disease.

Prorenin levels are increased in the plasma and kidney in diabetes^64–66^. In the present study, in rats with STZ-induced hyperglycemia, renin content was augmented in the plasma and prorenin and renin amounts were increased in the renal medullary tissues. These findings further support the concept that in this rat model, concomitant augmented excretion of renin and Ang II contents in the urine further supports the concomitant activation of the intratubular RAS. Further studies are ongoing to examine the relative contribution of other RAS components to this mechanism.

The PRR exhibits a full-length molecular form that is bound to the PM and a soluble form that is secreted into the extracellular space, including plasma^33^. urine^27^, and extracellular media^67,68^. In the STZ-induced hyperglycemic rats of the present study, sPRR levels were decreased in plasma, but in contrast, it was augmented in the urine compared with those levels quantified in controls, suggesting that sPRR in the urine does not come from the systemic circulation. We previously demonstrated that augmented sPRR in the urine of chronic Ang II-infused rats is associated with increased renin activity in the urine despite the characteristic suppression of PRA in these hypertensive rats^27^. Nonetheless, it remains unclear if renal intratubular sPRR could actually contribute to the activation of prorenin locally secreted by the CD. The sPRR is generated by the cleavage action of furin, ADAM 19, and S1P^32,33,69^. Furin expression is augmented in glomerular cells in HG conditions^70,71^; however, in the present study, intrarenal furin mRNA levels did not differ between two groups of rats (Fig. 5C). Protein levels were also examined; however, we did not detect differences among the groups (Fig. 5D). It is possible that this protease is not the primary source of sPRR augmentation in the urine of STZ hyperglycemic rats, therefore, the involvement of other intracellular serine proteases should be explored.

The present study highlights the effects of HG on PRR in M-1 cells showing the responses at transcriptional, post-transcriptional and trafficking levels. Luo et al., recently reported that HG caused PRR degradation by autophagy and ubiquitin–proteasome in rat inner medullary CD cells^65^. These effects were accompanied by upregulation of PRR mRNA, but downregulation of its protein levels. Pre-treatment with proteasome inhibitor MG-132 for 4 h blunted these effects and increased PRR protein levels after 3 d of cell exposure to HG^65^. Discrepancies between the two studies could be due to the differences between the two cell models used. We performed the experiments using M-1 cells, an established cortical CD cell line from mouse origin; while Siragy and associates employed primary cell cultures from rat renal inner medulla. Nevertheless, it is important to carefully address the deleterious effects that HG may exert on cells of the distal nephron segment, which usually are not exposed to luminal glucose. Increased PRR abundance in the CD might be twice harmful. On one side, it results in increases in renin activity and intratubular Ang II formation^9,28,29^, if renal AGT and ACE activity, are present. On the other side, it triggers intracellular signals that upregulate pro-fibrotic factors^12,48,52^. Therefore, during HG conditions as occurs in diabetes, PRR in the CD may contribute to increasing not only sodium reabsorption by Ang II-dependent mechanisms, but also to tubulointerstitial fibrosis independent of Ang II generation. Further studies are encouraged to examine the underlying mechanisms involved in the HG-dependent regulation of PRR.

In conclusion, our data suggest that the presence of high glucose in the distal nephron segments may induce PRR translocation to the apical cell side in the CD cells. During these conditions, high glucose facilitates the physical interaction between PRR and prorenin, thereby increasing PRR-dependent upregulation of downstream fibrotic factors, tubulointerstitial fibrosis, and likely, other inflammatory cytokines as TNF-α and IL-1β, independent of renal Ang II^58^. Our data may also serve as a trigger for future investigations considering PRR blockade as a novel target for the prevention and progression of kidney disease in patients with diabetes.

## Supplementary Information

Supplementary Information.
