# Supplementary material for: High glucose induces trafficking of prorenin receptor and stimulates profibrotic factors in the collecting duct
Source: Sci Rep. 2021 Jul 5;11:13815. doi: 10.1038/s41598-021-93296-4 (PMC8257763; doi:10.1038/s41598-021-93296-4)

Blots band identity

High glucose induces trafficking of prorenin receptor and stimulates profibrotic factors in the collecting duct

Venkateswara R Gogulamudi[1], Danielle Y Arita[1], Camille R.T Bourgeois[1], Justine Jorgensen[1], Jing He[2], William C. Wimley[2], Ryousuke Satou[1], Alexis A. Gonzalez[3] and Minolfa C Prieto[1,4].

[1]Department of Physiology, Tulane University, School of Medicine, New Orleans, LA; [2]Department of Biochemistry, Tulane University School of Medicine; [3]Instituto de Química, Pontificia Universidad Católica de Valparaíso, Valparaiso, Chile; and. [4]Hypertension and Renal Center of Excellence, Tulane University School of Medicine, New Orleans, LA-USA.

Blot Figure 2 renin and prorenin n=4

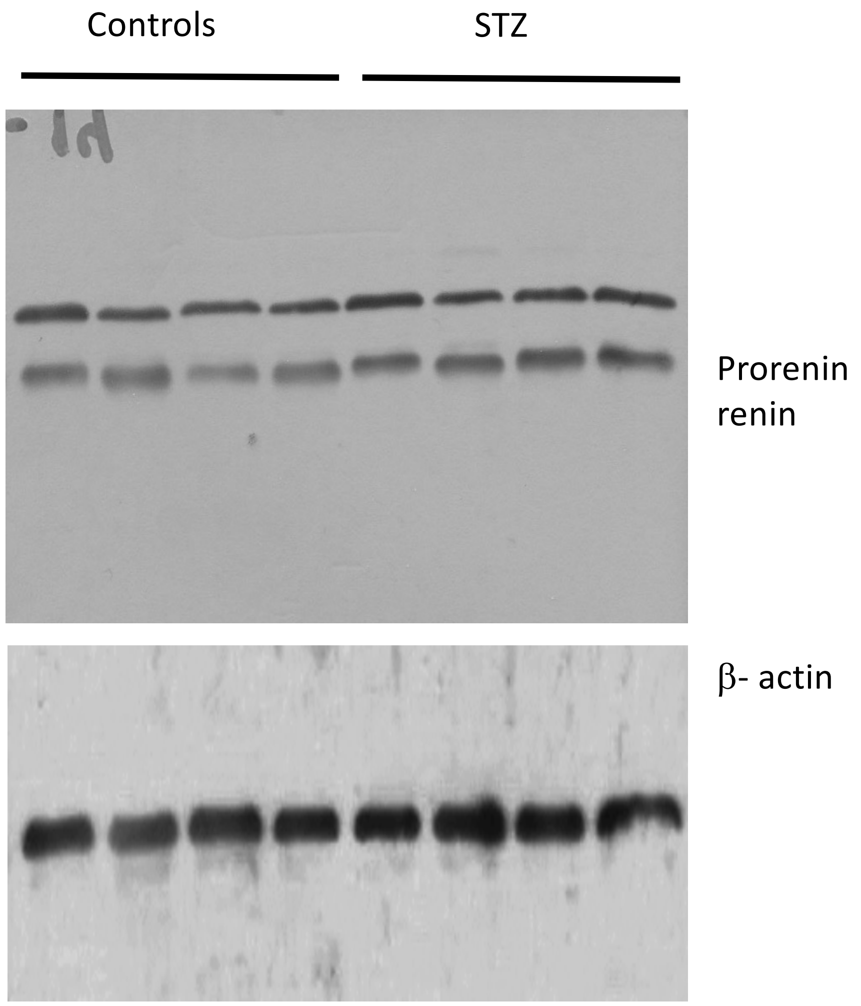

Figure 4 renin and prorenin prorenin receptor n=3

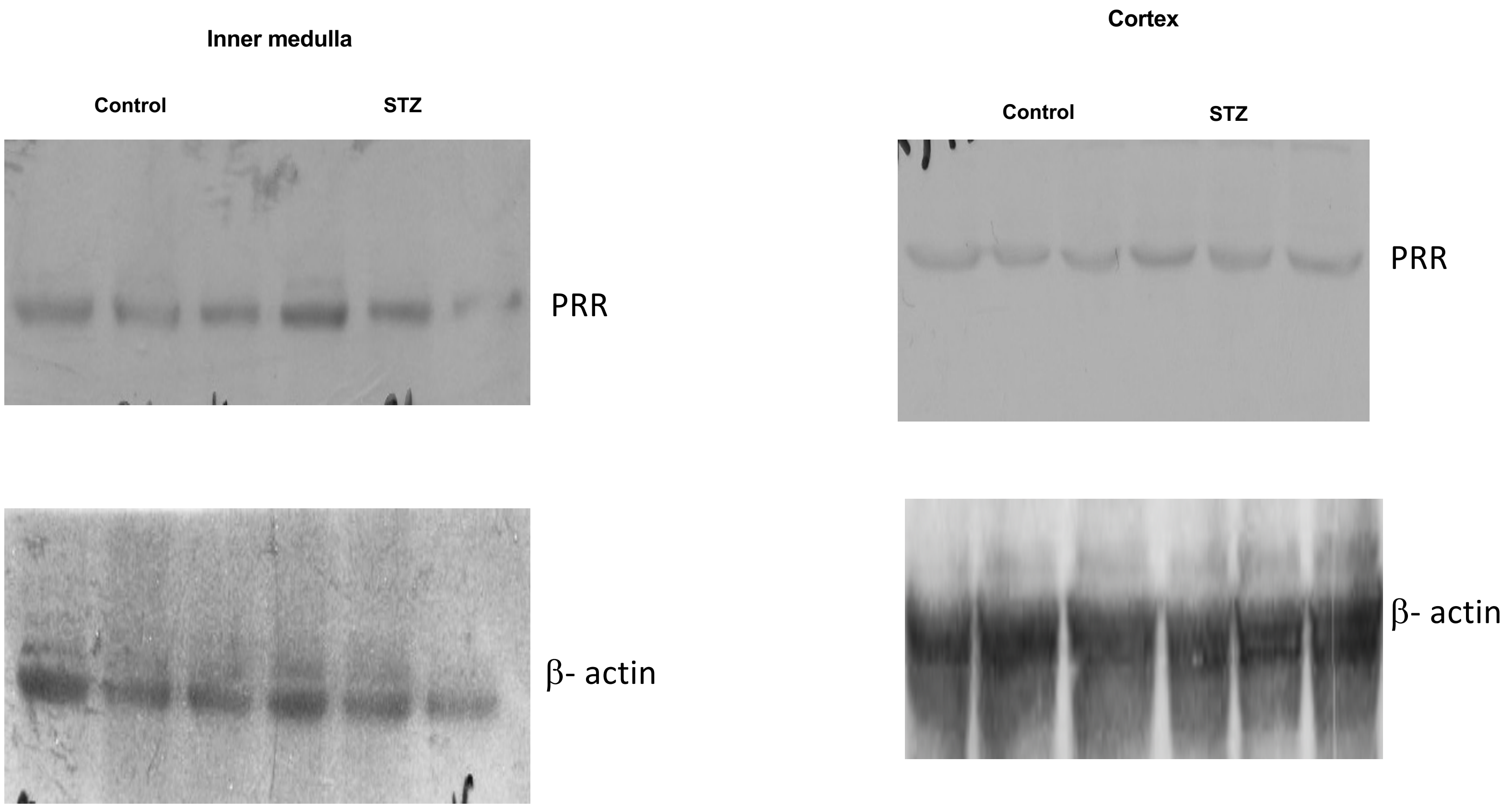

Figure 5 furin protein n=4

Furin –

-50 kDa

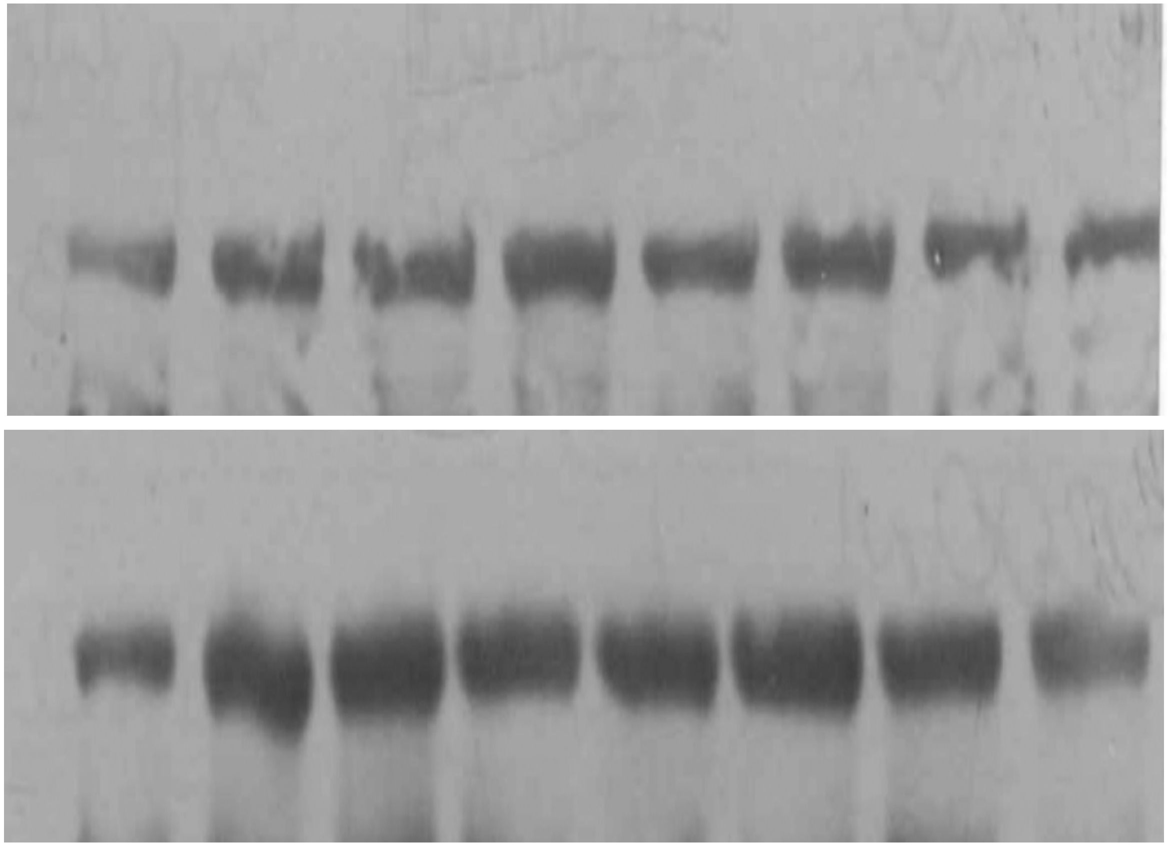

-40 kDa

β -actin -

FIGURE 6

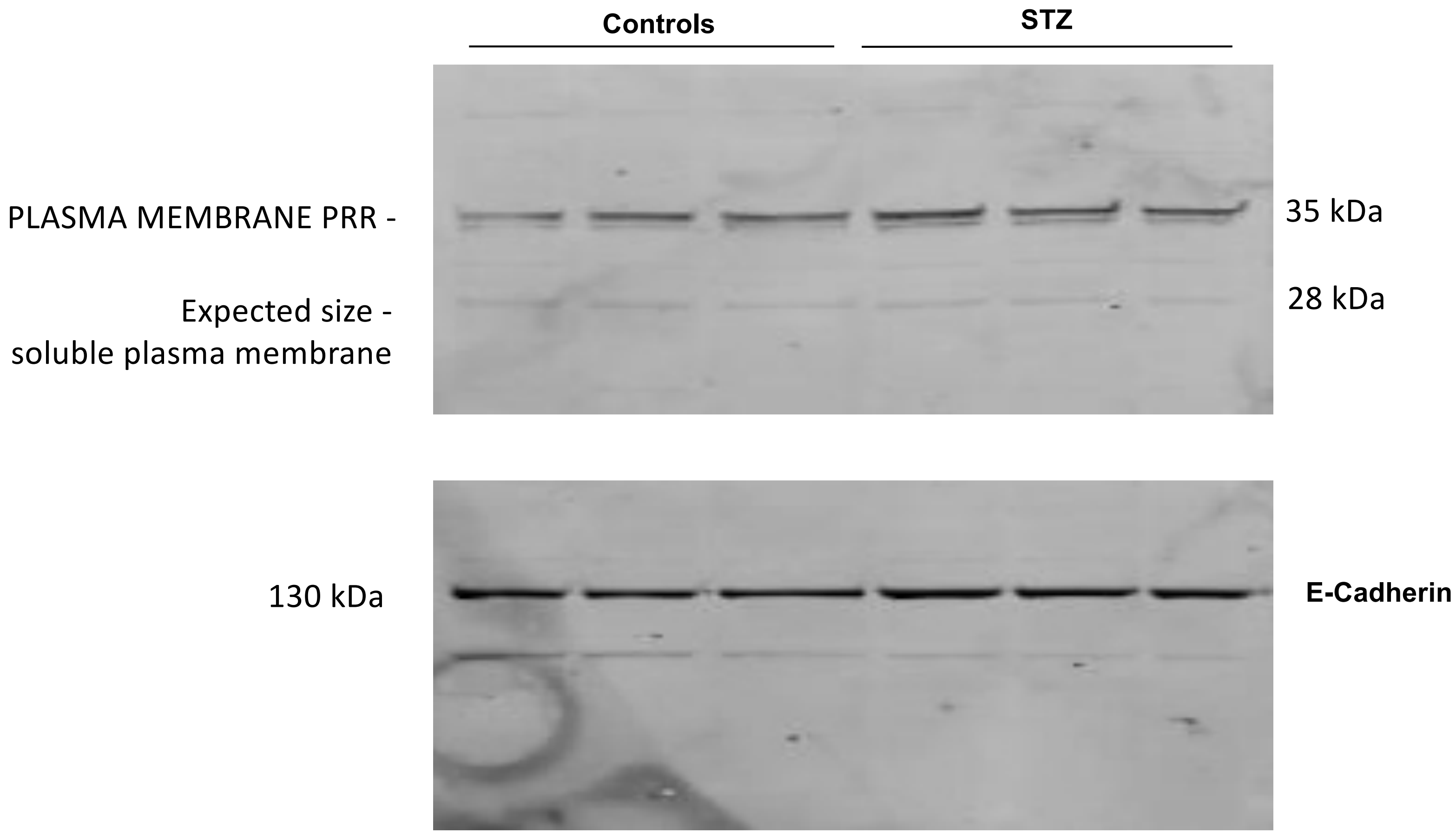
Controls
STZ
PLASMA MEMBRANE PRR -
35 kDa
Expected size -
soluble plasma membrane
28 kDa
130 kDa
E-Cadherin

FIGURE 8

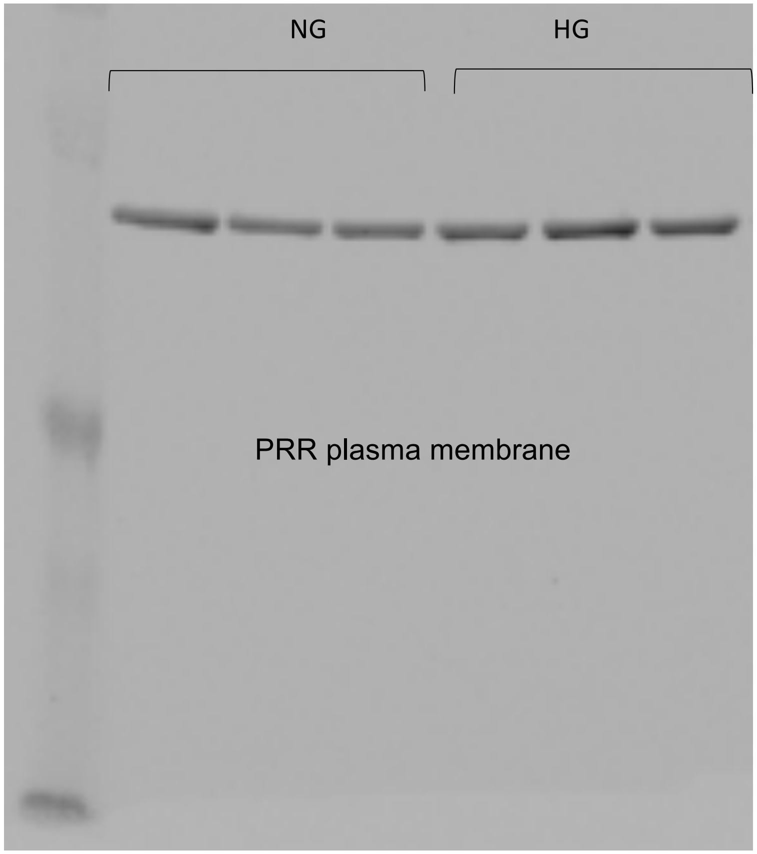

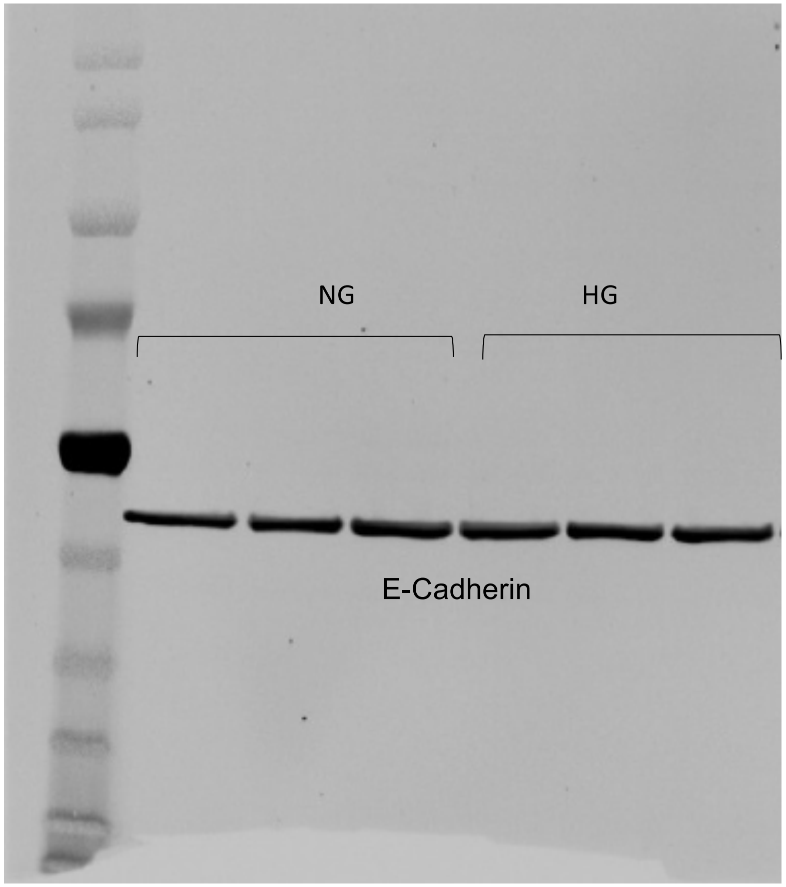

FIGURE 8

RENIN AND PRORENIN IN CULTURE MEDIA 10 X CONTRATED

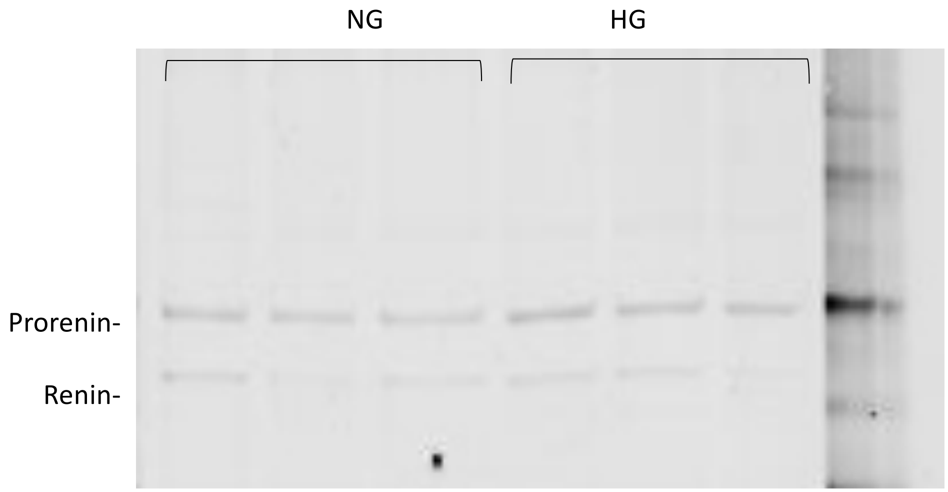

FIGURE 9 A

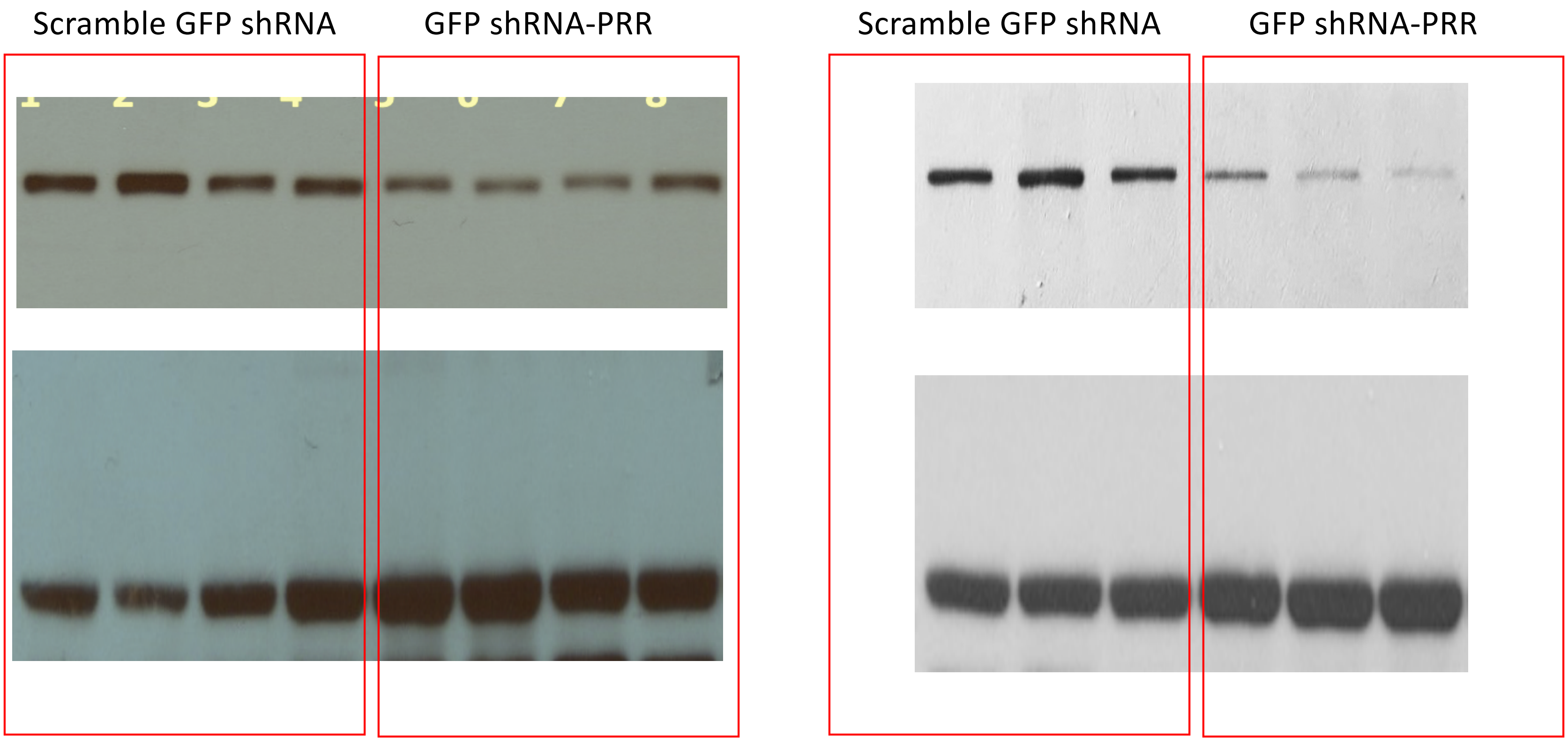
Scramble GFP shRNA
GFP shRNA-PRR
Scramble GFP shRNA
GFP shRNA-PRR

FIGURE 9 B

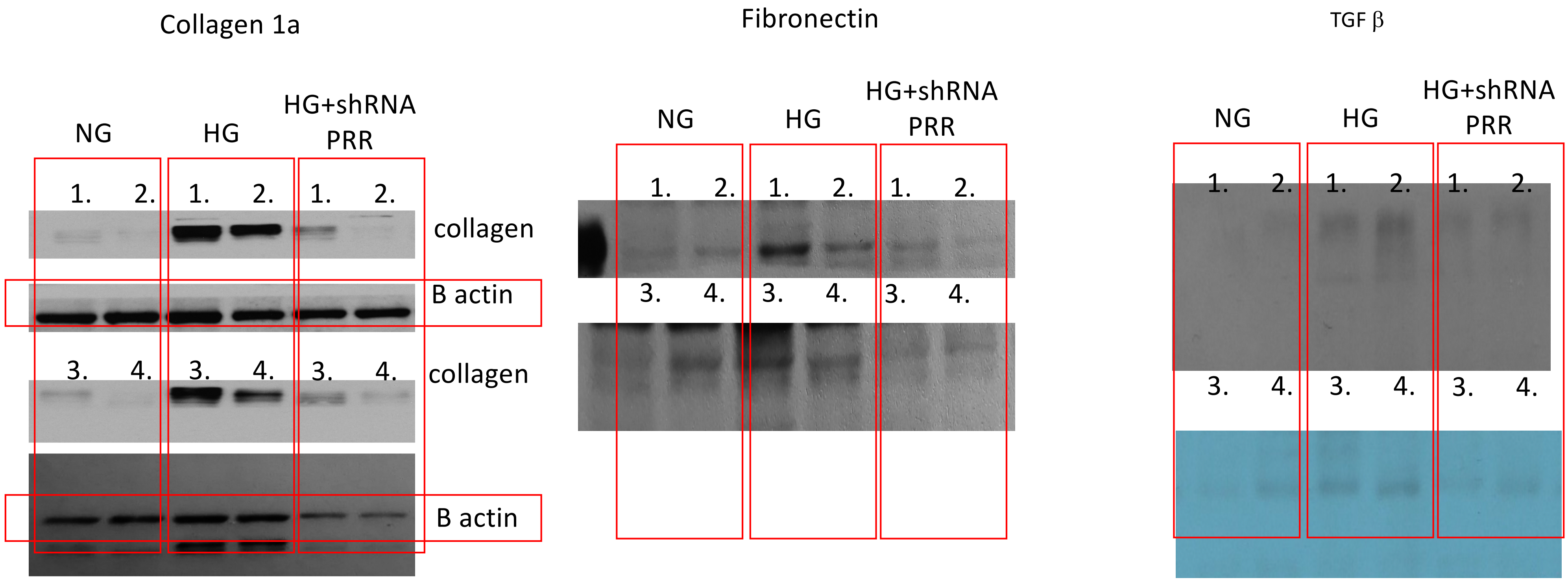
Collagen 1a
NG
HG
HG+shRNA PRR
1. 2. 1. 2. 1. 2.
collagen
B actin
3. 4. 3. 4. 3. 4.
collagen
B actin
Fibronectin
NG
HG
HG+shRNA PRR
1. 2. 1. 2. 1. 2.
3. 4. 3. 4. 3. 4.
TGF β
NG
HG
HG+shRNA PRR
1. 2. 1. 2. 1. 2.
3. 4. 3. 4. 3. 4.

FIGURE 9 C

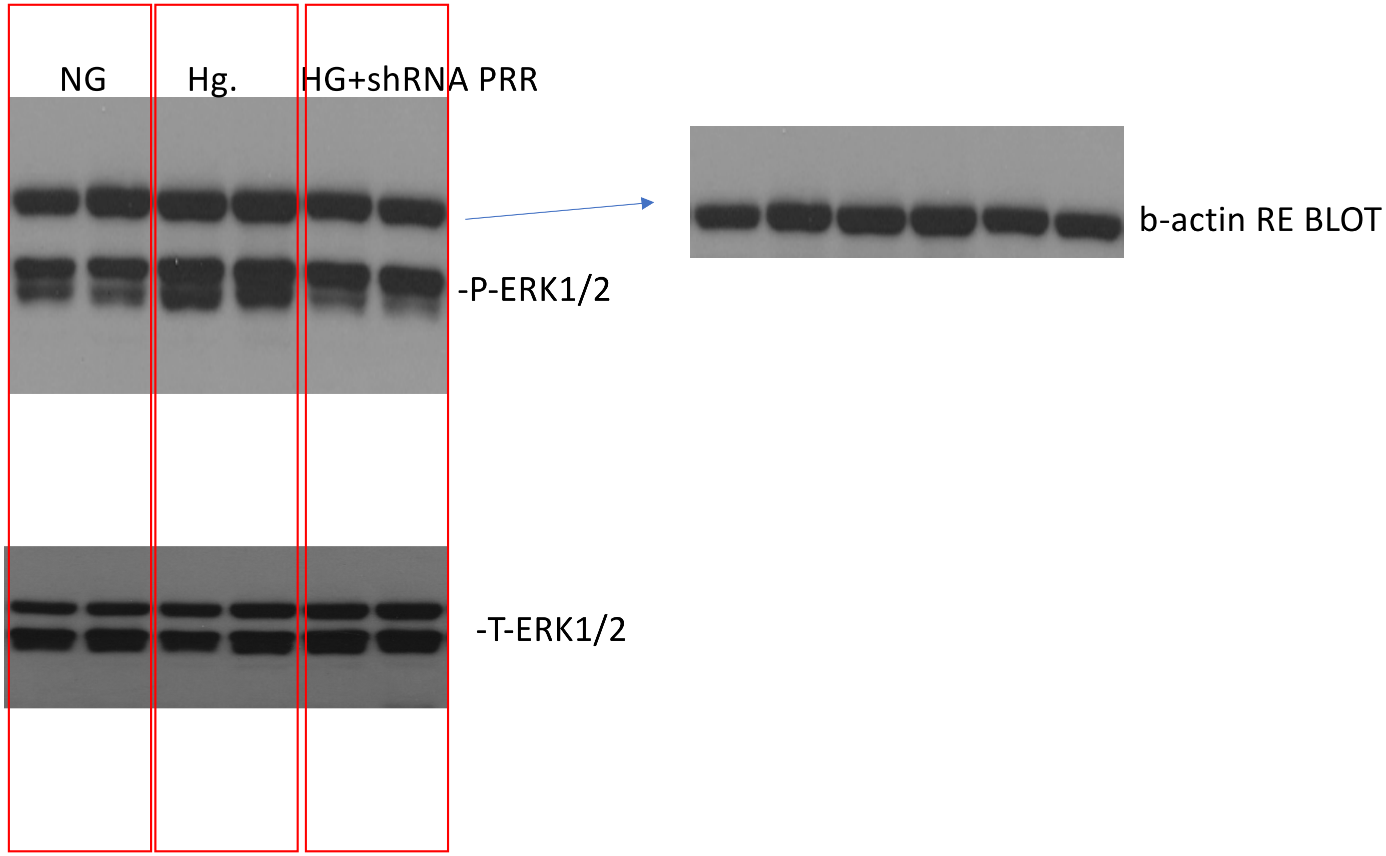

Supplement: Supplementary file 1 — Supplementary Information. [file 41598_2021_93296_MOESM1_ESM.pdf]
